# Developmental exposures to common environmental contaminants, DEHP and lead, alter adult brain and blood hydroxymethylation in mice

**DOI:** 10.3389/fcell.2023.1198148

**Published:** 2023-06-13

**Authors:** Rebekah L. Petroff, Raymond G. Cavalcante, Justin A. Colacino, Jaclyn M. Goodrich, Tamara R. Jones, Claudia Lalancette, Rachel K. Morgan, Kari Neier, Bambarendage P. U. Perera, Christine A. Rygiel, Laurie K. Svoboda, Kai Wang, Maureen A. Sartor, Dana C. Dolinoy

**Affiliations:** ^1^ Department of Environmental Health Sciences, University of Michigan School of Public Health, Ann Arbor, MI, United States; ^2^ Epigenomics Core, Biomedical Research Core Facilities, Michigan Medicine, Ann Arbor, MI, United States; ^3^ Department of Nutritional Sciences, University of Michigan School of Public Health, Ann Arbor, MI, United States; ^4^ Department of Pharmacology, University of Michigan Medical School, Ann Arbor, MI, United States; ^5^ Department of Computational Medicine and Bioinformatics, Michigan Medicine, Ann Arbor, MI, United States; ^6^ Department of Biostatistics, University of Michigan School of Public Health, Ann Arbor, MI, United States

**Keywords:** DNA methylation, DNA hydroxymethylation, lead (Pb), phthalate, DEHP (di-(2-ethylhexyl) phthalate), toxicoepigenetics, 5-hydroxymethylcytosine

## Abstract

**Introduction:** The developing epigenome changes rapidly, potentially making it more sensitive to toxicant exposures. DNA modifications, including methylation and hydroxymethylation, are important parts of the epigenome that may be affected by environmental exposures. However, most studies do not differentiate between these two DNA modifications, possibly masking significant effects.

**Methods:** To investigate the relationship between DNA hydroxymethylation and developmental exposure to common contaminants, a collaborative, NIEHS-sponsored consortium, TaRGET II, initiated longitudinal mouse studies of developmental exposure to human-relevant levels of the phthalate plasticizer di(2-ethylhexyl) phthalate (DEHP), and the metal lead (Pb). Exposures to 25 mg DEHP/kg of food (approximately 5 mg DEHP/kg body weight) or 32 ppm Pb-acetate in drinking water were administered to nulliparous adult female mice. Exposure began 2 weeks before breeding and continued throughout pregnancy and lactation, until offspring were 21 days old. At 5 months, perinatally exposed offspring blood and cortex tissue were collected, for a total of 25 male mice and 17 female mice (*n* = 5–7 per tissue and exposure). DNA was extracted and hydroxymethylation was measured using hydroxymethylated DNA immunoprecipitation sequencing (hMeDIP-seq). Differential peak and pathway analysis was conducted comparing across exposure groups, tissue types, and animal sex, using an FDR cutoff of 0.15.

**Results:** DEHP-exposed females had two genomic regions with lower hydroxymethylation in blood and no differences in cortex hydroxymethylation. For DEHP-exposed males, ten regions in blood (six higher and four lower) and 246 regions (242 higher and four lower) and four pathways in cortex were identified. Pb-exposed females had no statistically significant differences in blood or cortex hydroxymethylation compared to controls. Pb-exposed males, however, had 385 regions (all higher) and six pathways altered in cortex, but no differential hydroxymethylation was identified in blood.

**Discussion:** Overall, perinatal exposure to human-relevant levels of two common toxicants showed differences in adult DNA hydroxymethylation that was specific to sex, exposure type, and tissue, but male cortex was most susceptible to hydroxymethylation differences by exposure. Future assessments should focus on understanding if these findings indicate potential biomarkers of exposure or are related to functional long-term health effects.

## 1 Introduction

In early development, embryonic and fetal programming orchestrates finely tuned processes that help establish long-term health and wellbeing. During this time, adverse events, such as exposure to environmental contaminants (Heindel et al, 2015), may disrupt these processes, leading to a higher risk of health effects later in life. This hypothesis, called the Developmental Origins of Health and Disease (DOHaD) (Barker, 2007), has been widely studied in epidemiological and animal models. The biological processes behind the hypothesis, however, are poorly understood.

One process that may underly the DOHaD hypothesis is disruption to early epigenetic programming (Barouki et al, 2018). The epigenome can be defined as mitotically (and sometimes meiotically) heritable marks that help regulate gene expression without altering the genome itself (Murrell et al, 2005). These can include marks on nucleotides (e.g., DNA methylation, hydroxymethylation), histone modifications (e.g., acetylation, phosphorylation, ubiquitination), or noncoding RNA molecules, such as long noncoding RNA (lncRNA) (Greally, 2018). One of the most studied epigenetic marks is DNA methylation. DNA methylation is established early in life. In development, the ova and sperm methylome are erased immediately after fertilization and gradually rewritten throughout gestation (Monk et al, 1987). A secondary phase of fetal programming takes place during primordial germ cell development and migration (Seisenberger et al, 2012). The writing of DNA methylation occurs via a cyclical process: first methylation marks are added to the nucleotides (often cytosines upstream of guanines, CpGs, when methylated, 5-mC) by DNA methyltransferases (DNMTs). Methyl marks can then be oxidized to hydroxymethylation (5-hydroxymethylcytosine, or 5-hmC) by ten-eleven translocation (TET) dioxygenases (Tahiliani et al, 2009), which can be further modified to 5-formylcytosine (5-fC) and 5-carboxylcytosine (5-caC). Both 5-fC and 5-caC can be removed and replaced with a naked cytosine via base excision repair (Moore et al, 2013).

DNA methylation and hydroxymethylation are stable and present throughout tissues in the body (Globisch et al, 2010; Li and Liu, 2011; Wu et al, 2011; Nestor et al, 2012) and likely have opposing regulatory effects on gene expression (Wu and Zhang, 2017). Traditionally, DNA methylation has been thought to have a gene “silencing” effect, but the exact effects are dependent on where the modifications are in the genome (Jones, 2012). Hydroxymethylation plays an essential role in normal development (Yan et al, 2023), helping regulate development in both the heart (Greco et al, 2016) and brain (Stoyanova et al, 2021). Especially in promotor regions, it may reverse the effects of methylation (Mellén et al, 2017). Throughout the genome, it can also act as a recruiter and signal for other epigenetic factors (Takai et al, 2014). While methylation and hydroxymethylation are inherently linked, the developmental programming of these marks do have some independence (Amouroux et al, 2016; Lopez et al, 2017; Yan et al, 2023). However, most environmental exposure studies evaluating the methylome and hydroxymethylome do not use methods that differentiate between these marks; instead, studies typically report on combined “total methylation” (Booth et al, 2012). Because adverse events during these processes could disrupt programming and alter the methylome and hydroxymethylome independently, it is essential to understand the unique response of each of these epigenetic marks.

Some environmental contaminants, such as the group of plasticizers known as phthalates, have developmental effects that have been recently identified. Exposure to phthalates is nearly universal (Woodruff et al, 2011; Zota et al, 2014) and has been linked with endocrine disrupting effects and an increased risk of metabolism and neurodevelopmental disorders and diseases (Braun, 2017). Other common contaminants, such as the metal lead (Pb), have been widely known as developmental toxicants for decades. Early life exposure to Pb can occur via drinking water, contaminated soil, or dust, which can subsequently disrupt brain development, slow growth, and impact the immune system (Bellinger et al, 2017). In animal models, other contaminants, like the plastic additive bisphenol A (BPA) may cause changes in hydroxymethylation (Kochmanski et al, 2018). Developmental exposure to both phthalates (Svoboda et al, 2020; Hsu et al, 2021; Liu et al, 2021) and Pb (Dou et al, 2019b; Wang et al, 2020; Hong et al, 2021; Svoboda et al, 2021) have been linked to differences in the epigenome-wide total methylation, but little is known about effects in the hydroxymethylome.

To understand differences in the hydroxymethylome after developmental exposure to both the phthalate, di(2-ethylhexyl) phthalate (DEHP), and Pb, a longitudinal mouse model was used. This study was conducted as a part of the National Institute of Environmental Health Sciences (NIEHS) Toxicant Exposures and Responses by Genomic and Epigenomic Regulators of Transcription II (TaRGET II) Consortium, which aims to determine how the environment affects disease susceptibility across the life course through changes to the epigenome. We hypothesized that perinatal DEHP and Pb exposure would result in tissue- and sex-specific changes in DNA hydroxymethylation in adulthood.

## 2 Materials and methods

### 2.1 Study Design

Wild-type non-agouti *a/a* mice were obtained from a 230+ generation colony of agouti viable yellow (*A* ^*vy*^) mice, which are genetically invariant and 93% identical to C57BL/6J mice (Dou et al, 2019a). Virgin *a/a* females (6–8 weeks old) were randomly assigned to one of three exposure groups: 5 mg DEHP/kg chow/day, 32 ppm Pb-acetate drinking water, or control (DEHP- and Pb-free). Exposure began 2 weeks prior to mating with virgin *a/a* males (7–9 weeks old) and continued until offspring weaning at postnatal day 21 (PND 21). Animals were maintained on phytoestrogen-free modified AIN-93 chow (Envigo Td.95092, 7% corn oil diet, Harlan Teklad) and housed in polycarbonate-free cages. All procedures were approved by the University of Michigan Institutional Animal Care and Use Committee (IACUC) and conducted in accordance with experimental procedures outlined by the NIEHS TaRGET II Consortium and the highest animal welfare standards (Wang et al, 2018).

**FIGURE 1:**
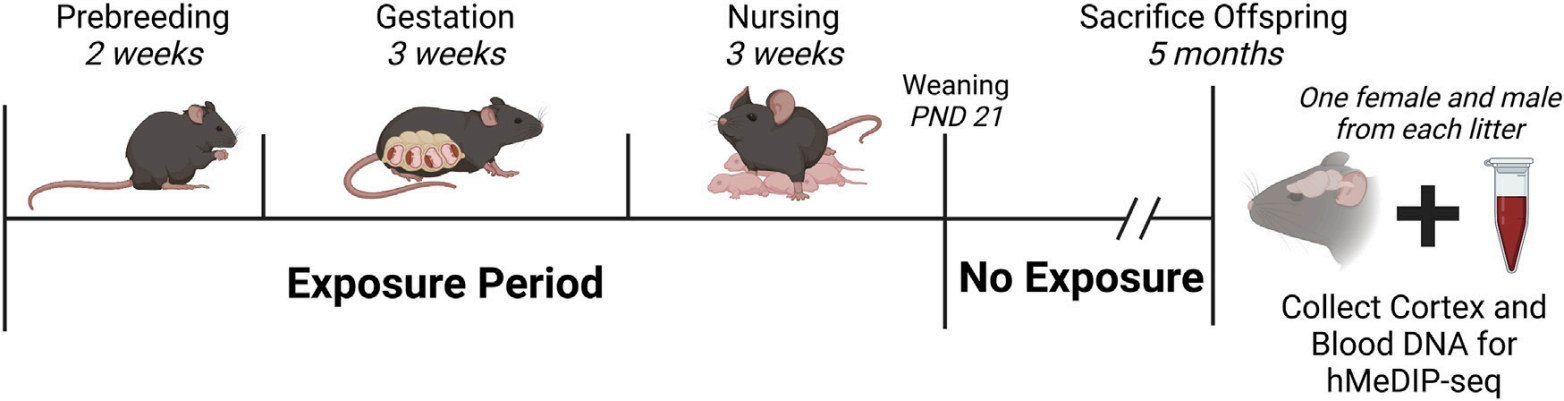
Study Design and Timeline. Nulliparous *a/a* mice were exposed to either 25 mg DEHP/kg food chow, or 32 ppm Pb-acetate in drinking water, or assigned to a control group. Exposures began 2 weeks before breeding, continued throughout pregnancy/gestation and lactation, and ceased at weaning (PND 21). At 5 months, one female and one male from each litter were sacrificed, and cortex and blood were collected for DNA hydroxymethylation analysis. Final sample sizes were: DEHP-females, *n* = 5; DEHP-males, *n* = 7 for cortex, *n* = 6 for blood; Pb-females, *n* = 6; Pb-males, *n* = 6; control-females, *n* = 6; control-males, *n* = 6. Created in Biorender. Abbreviations: DEHP, di(2-ethylhexyl) phthalate; hMeDIP-seq, hydroxymethylated DNA immunoprecipitation sequencing; Pb, lead; PND, postnatal day.

DEHP and Pb exposures were conducted *ad libitum*. DEHP was dissolved in corn oil and used to create a 7% corn oil for chow. Assuming pregnant and nursing female mice weigh roughly 25 g and eat, on average, 5 g of chow per day, the resulting exposure level of 5 mg DEHP/kg bodyweight per day reflects human relevant exposures (Chang et al, 2017). Pb-acetate drinking water was prepared with distilled drinking water, with a concentration of 32 ppm to model human-relevant perinatal exposure. In previous work, we identified that this dose generates maternal blood levels (BLLs) ranging from 16 to 60 μg/dL (mean: 32.1 μg/dL) (Faulk et al, 2013). Individual animal exposures were not measured in the present study. At PND 21, all offspring were weaned and moved to either DEHP-free control chow or Pb-free control water and maintained until 5 months of age (Figure 1).

### 2.2 Tissue collection and DNA extraction

Immediately following euthanasia with CO_2_ asphyxiation, blood was collected via cardiac puncture. Cortex tissue was dissected and immediately flash frozen in liquid nitrogen and stored at −80°C. The final sample size for this study included n ≥ 5 males and n ≥ 5 females in each exposure group (DEHP-exposed, Pb-exposed, and control) with 1 male and 1 female per litter per group. The final sample size for this study was n = 71, once tissues (i.e., cortex and blood) were collected. The AllPrep DNA/RNA/miRNA Universal Kit (Qiagen, Cat. #80224) was used to extract DNA from blood and cortex tissue. Extracted DNA was stored at −80°C until further processing.

### 2.3 hMeDIP-seq

Sample quality was assessed using the Agilent TapeStation genomic DNA kit (Agilent) and concentrations were measured using Qubit broad range dsDNA (Invitrogen). Ligation adapter arms were synthesized by IDT and hybridized in the University of Michigan Epigenomics Core Facility. Unless specified otherwise, the enzymes used for library preparation and dual-indexing primers were purchased from New England Biolabs.

For each sample, a total of 750 ng of genomic DNA was sheared by adaptive focused acoustics, using the Covaris S220 (Covaris). Sheared DNA was blunt-ended and phosphorylated. A single A-nucleotide was then added to the 3′ end of the fragments in preparation for ligation of adapter duplex with a T overhang. The ligated fragments were cleaned using Qiagen’s MinElute PCR purification columns. DNA standards for hMeDIP-seq (Diagenode, 5-hmC, 5-mC, & cytosine DNA standard pack for hMeDIP, cat # AF-107–0040) were added to each sample before denaturation and resuspension in ice-cold immunoprecipitation buffer (10 mM Sodium Phosphate pH 7.0, 140 mM NaCl, 0.05% Triton X-100). A 10% volume was input before 2 µg of a 5-hmC-specific antibody (Active Motif, Cat # 39791) was added for immunoprecipitation overnight at 4°C with rotation. Dynabeads Protein-G (Invitrogen) were added to the immunoprecipitation to perform the pull-down of 5-hmC-enriched fragments. The 5-hmC-enriched DNA fragments were then released from the antibody by digestion with Proteinase K (Ambion).

After cleanup with AMPure XP beads (Beckman Coulter), the percent input in the 5-hmC enriched fragments was evaluated by qPCR, using primers specific for the spike-ins. Samples with good percent input were then PCR amplified for the final library production, cleaned using AMPure XP beads, and quantified using the Qubit assay and TapeStation High Sensitivity D1000 kit. The libraries were pooled and then sequenced on a NovaSeq6000 instrument at the University of Michigan Advanced Genomics Core Facility.

### 2.4 Data processing and analysis

Reads were assessed for quality (FastQC v0.11.8), had adapter sequence trimmed (TrimGalore v0.4.5), and aligned to mm10 with Bowtie2 (v2.3.4.1) (Langmead and Salzberg, 2012) using default parameters (excepting -X 2000). Duplicate reads were marked with Picard (v2.20.2) and filtered out with samtools (v1.2) (Li et al, 2009). Alignments that overlapped ENCODE blacklisted regions were removed with bedtools (v2.28.0) (Quinlan and Hall, 2010) and the resulting reads were used for peak calling with macs2 (v2.1.2) (Zhang et al, 2008). Additional ChIP QC measures were determined with phantompeakqualtools (Landt et al, 2012) and DeepTools (v3.3.0) (Ramirez et al, 2016).

Analyses were conducted in R (v > 4.1) using Bioconductor packages (R Core Team, 2021). Sex, tissues, and exposures were analyzed separately. Using DiffBind (Stark and Brown, 2011; Ross-Innes et al, 2012), consensus peaks (overlapping in at least 66% of samples in each comparison group) were exacted. Individual consensus peaks were counted using 100 and 500 bp windows and normalized based on library size. Both 100 and 500 bp counts were analyzed using DESeq2 options, and regions with a significantly different count between exposed and control groups were identified, using a false-discovery rate (FDR) cutoff of 0.15. To minimize false positives, only regions with FDR<0.15 in both the 100 and 500 bp analyses were considered as true positives.

Regions were annotated to the mm10 genome using annotatr (Calvacante and Sartor, 2017). Random regions were generated and annotated to compare relative frequencies of annotations. For comparisons with more than 100 mapped genes on the differential regions, gene sets were assessed for gene ontology using ChIP-Enrich (Welch et al, 2014).

## 3 Results

### 3.1 Differential hydroxymethylation: DEHP

**FIGURE 2:**
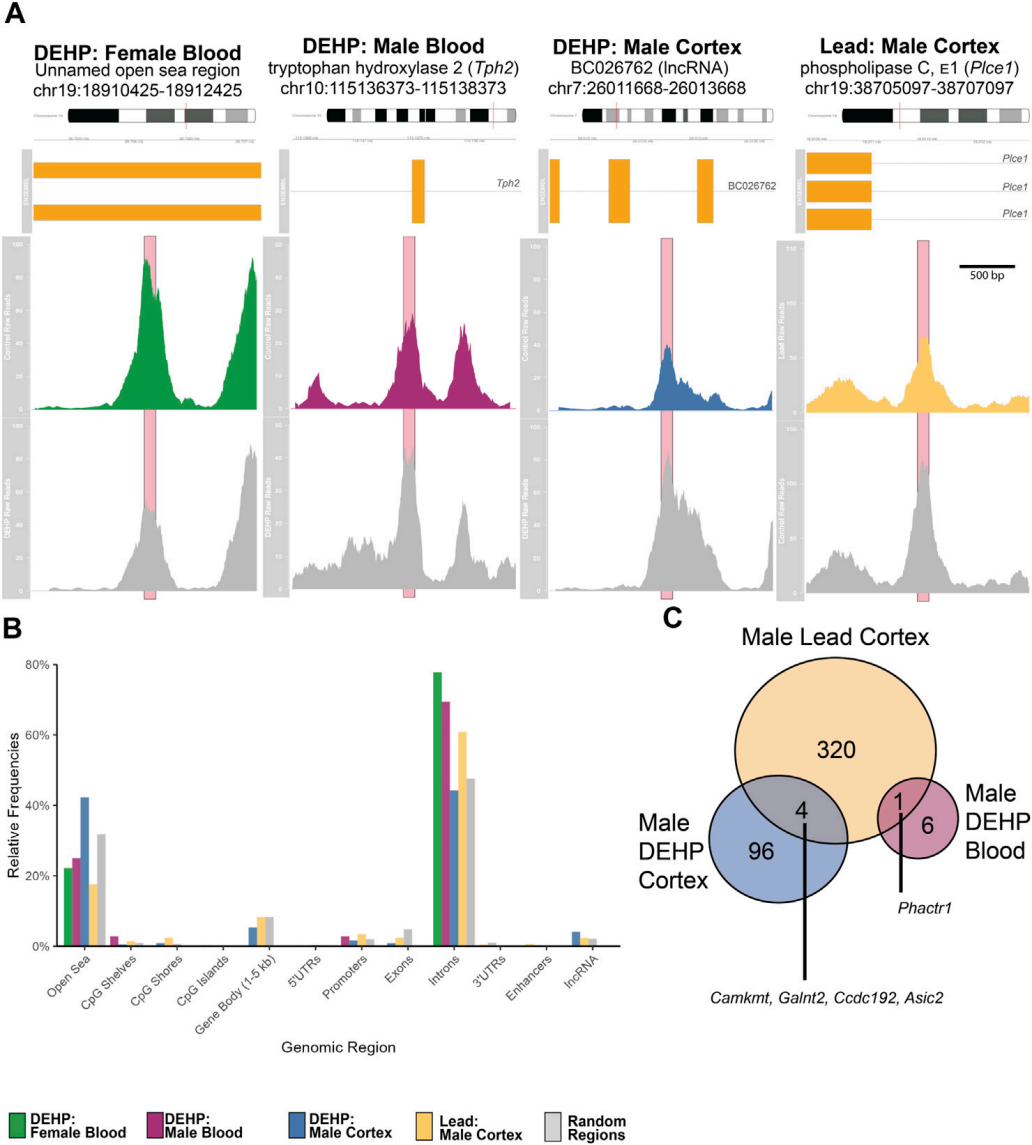
**(A)** Averaged Raw Reads from Top Hit in Each Comparison. Top colored panel in each panel represents the exposed group, bottom gray panel represents control. Significant peaks are marked with pink boxes. Gene region details show chromosomal and genomic location, as well as gene variants and exons (in yellow boxes). Each plot depicts the differentially hydroxymethylated region ± 1,000 bp. Detailed peak data is found in Supplementary Table. **(B)** Frequencies of Annotated Regions in Significant Peaks. Each bar shows the percentage of annotated peaks in that category using the annotatr package and mm10 genome in R. For each category, the left-most bar represents DEHP female blood peaks (green), second from left represents the DEHP male blood peaks (purple), the middle represents the DEHP male cortex peaks (blue), the second from the right represents the Pb male cortex peaks (yellow), and the right-most bar represents the randomly generated regions for comparisons (gray). **(C)** Venn Diagram of Annotated Differentially Hydroxymethylated Genes in Males. Unique genes that were overlapping in DEHP blood, DEHP cortex, and Pb (lead) cortex. No specific regions within those genes overlapped. There were no overlaps with female differentially hydroxymethylated regions. Abbreviations: CpG–cytosine-guanine site; DEHP–di(2-ethylhexyl) phthalate; lncRNA–long noncoding RNA; Pb–lead; UTR–untranslated region.

For females (n = 5 exposed, n = 6 control), there were two differentially hydroxymethylated regions (DhMRs) in 5-month blood comparing DEHP-exposed and control groups (Figure 2A; Table 1; Supplementary Table S1; Supplementary Figure S1A). In both regions, an intron region on the *Cit* gene and an open sea region on chromosome 19 were less hydroxymethylated in the DEHP group compared to the controls. These two regions or genes did not overlap with regions or genes from other comparisons (e.g., female DEHP cortices, male tissues, or any comparison from Pb exposures). There were no regions with differential hydroxymethylation in female cortex (n = 5 exposed, n = 6 control).

**TABLE 1:** Number of differentially hydroxymethylated regions by exposure (FDR<0.15).

Sex	Tissue	Higher in exposed	Lower in exposed	Total
DEHP				
Females	Blood	-	2	2
	Cortex	-	-	0
Males	Blood	6	4	10
	Cortex	242	4	246
Pb (Lead)				
Females	Blood	-	-	0
	Cortex	-	-	0
Males	Blood	-	-	0
	Cortex	385	-	385

For male blood (n = 6 exposed, n = 6 control), there were ten genomic regions in 5-month blood that had differential hydroxymethylation, with six regions higher in exposed compared to control and four regions lower in exposed (Figure 2A; Table 1; Supplementary Table S2; Supplementary Figure S1B). Nearly 70% of these DhMRs mapped to introns of genes (Figure 2B); lower hydroxymethylated regions included introns in *Fhod3*, *Fbxl12*, *Nos1*, and *Tmem266*. Higher hydroxymethylation in the DEHP-exposed male blood included regions in introns in *Tph2*, *Bace1*, and *Phactr1* and two unnamed, open sea regions on chromosomes 3 and 5. Only one annotated DhMR gene overlapped with any other comparison (e.g., male DEHP cortex, female DEHP blood, or any comparison from Pb exposures)—*Phactr1* was also identified in male cortices exposed to Pb (Figure 2C).

Adult male cortices developmentally exposed to DEHP (n = 7 exposed, n = 6 control) had 246 differentially hydroxymethylated regions, of which, only four had less hydroxymethylation in exposed **(** Figure 2A; Table 1; Supplementary Table S3; Supplementary Figure S1C). These regions mapped to 100 genes, with about 45% annotating to each gene introns and the open sea (Figure 2B **).** There were several regions that mapped to lncRNA, including *Macrod2os1*, *Mir99ahg*, *Gm26820*, *Gm15581*, *RP23-418H8.3*, *9130204K15Rik*, *4930511M06Rik/1700066O22Rik*, and *D930019O06Rik*. Other regions mapped to the promoters on *Kif2b*, *Slc12a7*, *Creg2*, and *Kctd19*. Of all 100 genes, there were four overlapping with the male Pb cortex genes, including *Camkmt*, *Galnt2*, *Ccdc192*, and *Asic2*. In mapped genes, gene ontology suggested that four pathways had differential hydroxymethylation levels in the male DEHP exposed cortices (Table 2). One pathway was a molecular function (solute and proton antiporter activity), and three pathways were biological processes in androgen receptor signaling and bone and biomineral regulation.

**TABLE 2:** Gene ontology results from annotated differentially hydroxymethylated regions (FDR<0.1).

Ontology name	GO ID	Type	Genes in GO Set (N)	Genes in Data (n)	*p*-value	FDR
DEHP (Male Cortex)						
solute:proton antiporter activity	GO:0015299	MF	15	2	1.20E-05	0.011
androgen receptor signaling pathway	GO:0030521	BP	45	4	1.06E-05	0.054
regulation of bone mineralization	GO:0030500	BP	71	6	3.35E-05	0.085
regulation of biomineral tissue development	GO:0070167	BP	79	6	5.18E-05	0.087
Pb (Male Cortex)						
postsynapse	GO:0098794	CC	460	26	1.26E-04	0.044
postsynaptic density	GO:0014069	CC	231	16	2.30E-04	0.044
postsynaptic specialization	GO:0099572	CC	233	16	2.71E-04	0.044
asymmetric synapse	GO:0032279	CC	234	16	3.24E-04	0.044
neuron to neuron synapse	GO:0098984	CC	236	16	3.66E-04	0.044
nuclear periphery	GO:0034399	CC	110	7	8.75E-04	0.088

Gene ontology (GO) results using differential hydroxymethylated regions from the male cortex and ChIP-Enrich in R. All pathways were upregulated in exposed groups. Abbreviations: DEHP, di(2-ethylhexyl) phthalate; BP, biological processes; CC, cellular component; MF, molecular function; Pb, lead.

### 3.2 Differential hydroxymethylation: Pb

For females (n = 6 exposed, n = 6 control for both blood and cortex), there were no differences of hydroxymethylation in either blood or cortices when comparing exposed to control groups across both blood and cortex.

For males (n = 6 exposed, n = 6 control for both blood and cortex), there were no differences in blood hydroxymethylation, but there were 385 regions that had universally higher hydroxymethylation in Pb-exposed cortex compared to controls **(** Figure 2A; Table 1; Supplementary Table S4; Supplementary Figure S1D). These DhMRs mapped to 325 unique genes, with annotations primarily in introns (∼18%) and open sea regions (∼60%) (Figure 2B). There were several DhMRs in lncRNA regions, including *Rian*, *Trerf1*, *Pvt1*, *Gm3294*, *Gm13575*, *Gm38190*, *Gm27247*, *Gm12278*, *Gm29295*, *Gm26883*, *Gm26904*, *Gm17435*, *Gm26691*, *Gm16183*, *RP23-304A10.2*, *RP23-363M4.1*, *2610203C22Rik*, *E130304I02Rik*, *2810407A14Rik*, *2610037D02Rik*, and *F630040K05Rik*. Regulatory regions were also identified in promoters for calcium related genes (*Cacnb4*, *Camk2g*, and *Cabp1*), genes that interact with DNA or epigenetic processes (*Mxi1*, *Mn*t, *Mthfd2*, *Ldb1*, *Mfrp*, *Hnrnpk*, *mir7074*, and *Yeats2*), an imprinted gene (*Rian*), an oncogene related to AP1 transcription factor complex (*Jund*), and several other genes related to various cell functions (*Gpr156*, *Ttll6*, *Paip1*, *Marcksl1*, *Kcnq2*, *Septin8*, *Dxd18*, *Dhx37*, *Tmco1*, and *4833412C05Rik*). There were also 12 known enhancer regions on chromosomes 1, 4, 7, 9, 11, 15, 17, and 18 annotated to differentially hydroxymethylated regions. Gene ontology of the 325 genes suggested that pathways related to neuronal and neural function were the primary pathways that showed differential hydroxymethylation (Table 2).

Comparing log-fold differences in hydroxymethylation between regions in the Pb-male cortex and analogous regions in the DEHP-male cortex, sites were poorly correlated (Supplementary Figure S2).

## 4 Discussion

While the epigenome is comprised of many different modifications and molecules, there has been a strong focus on DNA methylation in studies on the effects of exposures to common chemicals. However, these studies typically use methods that do not differentiate between types of DNA modifications, even though methylation and hydroxymethylation act in biologically opposite ways. In this study, we found that the genome-wide mouse hydroxymethylome is affected by developmental exposure to both DEHP and Pb, with differences in hydroxymethylation observed in adulthood.

In the brain, hydroxymethylation accounts for 33%–50% of DNA modifications, a much higher proportion than in other tissues (Cui et al, 2020). This high occurrence may indicate its importance in normal brain function, such as memory formation (Kremer et al, 2018). Brain hydroxymethylation also plays a role in response to injury (Morris-Blanco et al, 2019; Madrid et al, 2021; Moyon et al, 2021) and oxidative stress (Delatte et al, 2015), likely in a region-specific manner (Doherty et al, 2016). Its role in injury and oxidative stress responses may also be why hydroxymethylation across the body appears to be more sensitive to environmental assaults than other epigenetic marks (Chatterjee et al, 2020).

Exposure to DEHP or other phthalates has been associated with differences in bulk measures of hydroxymethylation in both human urine (Pan et al, 2016) and rat testes (Abdel-Maksoud et al, 2015). Presently, we found that perinatal exposure to DEHP was associated with later-in-life differences in region-specific blood hydroxymethylation in males and females and brain hydroxymethylation in males. There were much fewer regional differences in blood compared to brain, which could be related to the brain’s high levels of hydroxymethylation compared to other tissues. Perinatal DEHP exposure may also alter hydroxymethylation in a tissue specific manner, like with total DNA methylation in our same model (Wang et al, 2020; Liu et al, 2021; Svoboda et al, 2021). In the male brain, there were several regions in gene promoters responsible for microtubule control/cell division (*Kif2b*), cell transport (*Slc12a7*), neural-specific endoplasmic reticulum and Golgi functions (*Creg2*), and potassium channel function (*Kctd19*). These differences were also linked to gene ontology pathway enrichment in general cellular processes and in pathways related to androgen signaling and bone development. In humans, disruptions in androgen signaling have been one of the primary health effects of concern after developmental DEHP exposure. High exposures have been associated with decreased anogenital distances in males (Swan et al, 2005; Li and Ko, 2012) and long-term changes in growth and metabolism (Tsai et al, 2016; Tsai et al, 2018). More recently there have been concerns about bone growth and development in both animal models (Bielanowicz et al, 2016; Chiu et al, 2018) and humans (Heilmann et al, 2022).

Pb-exposed females showed no differences in hydroxymethylation; only Pb-exposed male brains had increases in hydroxymethylation in the brain. Hydroxymethylation differences after Pb exposure have been explored in various human tissues, including childhood blood (Rygiel et al, 2021), cord blood (Sen et al, 2015; Okamoto et al, 2022), toenail and placenta (Tung et al, 2022), and sperm (Zhang et al, 2021). Epigenome-wide differences were only assessed in toenails/placenta using the EPIC array (Tung et al, 2022) and in sperm using hMeDip-seq (Zhang et al, 2021). In toenails and placentas, most of the differentially hydroxymethylated sites were higher with Pb exposure (Tung et al, 2022), whereas sperm showed mostly lower hydroxymethylated regions (Zhang et al, 2021). Presently, all male cortex regions had higher hydroxymethylation with Pb exposure. All three epigenome-wide studies (Tung et al, 2022; Zhang et al, 2021, and the present study) identified differential hydroxymethylation in calcium genes or pathways. Because Pb is a bioanalogue of calcium, these similarities are expected. These three studies also consistently reported differences in pathways related to nervous system development and synapse function, even in non-neural tissues. This link should be further explored, as small, sparse, and local differences in hydroxymethylation have been associated with gene expression (Marion-Poll et al, 2022), potentially representing a “fine tuning” mechanism of gene regulation that is linked with transcription factor recruitment (Lercher et al, 2014). Because the brain is the primary target organ of Pb toxicity, collective results may be revealing differences in hydroxymethylation patterns that underlie the link between developmental Pb exposure and later life neurotoxic effects.

In Pb-exposed males, an imprinted gene (*Rian*) and several lncRNA genes were identified as differentially hydroxymethylated. Genomic imprinting is an epigenetically regulated process in which a gene is expressed from one allele in a parent of origin-specific manner. This phenomenon occurs in nearly 1% of the protein coding genes and includes maternally-expressed genes that are paternally imprinted, or vice versa (Barlow and Bartolomei, 2014; Kanduri, 2016). Imprinted genes are typically clustered to form imprinted domains, which also include the genetic code for at least 1-2 lncRNA. Imprinted domains often also contain gametic differentially methylated regions, one of which controls the entire domain to serve as an imprinting control region. The *Rian* lncRNA is part of a large imprinted domain on chromosome 12 (in mice). The human ortholog, *MEG8*, regulates vascular smooth muscle cell proliferation, migration, and apoptosis via miRNA interactions with the peroxisome proliferator activator receptor alpha (PPARα) (Zhang et al, 2019). Differential hydroxymethylation of imprinted genes was also identified in mice after developmental exposure to BPA (Kochmanski et al, 2018), which may confer broader patterns of disruption in imprinted genes after developmental exposure to common environmental contaminants.

While most identified regions were unique between exposures, there were four genes that overlapped between the DEHP and Pb in the male cortex, including *Camkmt*, *Galnt2*, *Ccdc192*, and *Asic2*. *Camkmt* encodes for a methyltransferase that assists in calcium dependent signaling, *Galnt2* encodes for a glycotransferase linked with metabolism functions, *Ccdc192* encodes for a long-noncoding RNA, and *Asic2* encodes for a protein in an ion channel, with high prevalence in the central nervous system. In the same cohort of mice from the present study, differential total methylation was identified in the *Galnt2* gene in heart tissue after DEHP and Pb exposure (Svoboda et al, 2021). Differential total methylation of the human ortholog of *Galnt2* was also identified in a human cohort exposed to Pb (Svoboda et al, 2021), representing either a potential biomarker of exposure or, due to the *Galnt2*’s large size, general epigenetic differences that broadly occur after environmental exposures. There was also one gene that overlapped in brain/blood across exposures–*Phactr1*, a gene that encodes for a phosphatase that regulates the actin cell structure. These overlapping genes may represent common areas of the hydroxymethylome that are particularly sensitive to environmental toxicants. Alternatively, as hydroxymethylation generally confers genome instability (Supek et al, 2014), these could be chance overlaps due to tertiary genome structure or other factors.

Hydroxymethylation is different between sexes and across ages, typically increasing throughout development (Cisternas et al, 2020). In our study, there were large differences between sexes, which may be a due to normal sex differences or due to sex differences in responses to environmental exposures. Additional studies with larger sample sizes and other model strains/species should be conducted, and the inclusion of physiological and behavioral endpoints should be emphasized, especially given the growing body of evidence on sex-specific responses to toxicants (Gade et al, 2021). Our smaller sample size could also result in false negatives. Validation studies with larger sample sizes should aim to include multiple developmental timepoints and tissues to understand hydroxymethylation differences in varying ages and across the body. Future validation studies could also consider PCR or nanopore techniques to confirm hMeDIP-seq results. Because we didn’t measure individual animal dietary intake of either DEHP or Pb, future studies should also measure toxicant exposures in offspring to better estimate dose-response relationships. Additionally in the brain, it is important to consider the impacts of non-cytosine hydroxymethylation (Ma et al, 2017), the parallel differences in methylation, and the cell-type specific patterns in epigenetic modifications which were not presently assessed.

Overall, this study demonstrates that sex-specific hydroxymethylation is different in adulthood in response to developmental environmental exposures, in not just the brain (specifically cortex), but also blood. The differences, however, were limited and should be validated with future work. Results were observed using a method that is able to detect regional differences across >95% of the genome, mostly in areas with lower cytosine-guanine base density (Beck et al, 2022). Because environmental epigenetic studies have traditionally only used methods that capture the collective DNA modifications (e.g., methylation + hydroxymethylation) in high density cytosine-guanine areas of the genome, many environmental effects in the hydroxymethylome may be masked. Future research needs to distinguish DNA methylation and hydroxymethylation functions and responses to environmental exposures, with the aim of revealing potential biomarkers or interventions in the exposure-disease pathway. The hydroxymethylome has had some promising studies in these areas already (Kim et al, 2018; Morris-Blanco et al, 2019; Morris-Blanco et al, 2021). Going forward, its role and responses to the environment should be a major research focus, sharing the spotlight with the diverse modifications and molecules of the epigenome.

## Data Availability

The data presented in the study are deposited in the NCBI Geo repository, accession number GSE229717.
