# Supplementary material for: Developmental exposures to common environmental contaminants, DEHP and lead, alter adult brain and blood hydroxymethylation in mice
Source: Front Cell Dev Biol. 2023 Jun 13;11:1198148. doi: 10.3389/fcell.2023.1198148 (PMC10294071; doi:10.3389/fcell.2023.1198148)
Supplement: Supplementary file 1 [file Presentation1.zip › Suppl. Figures.docx]

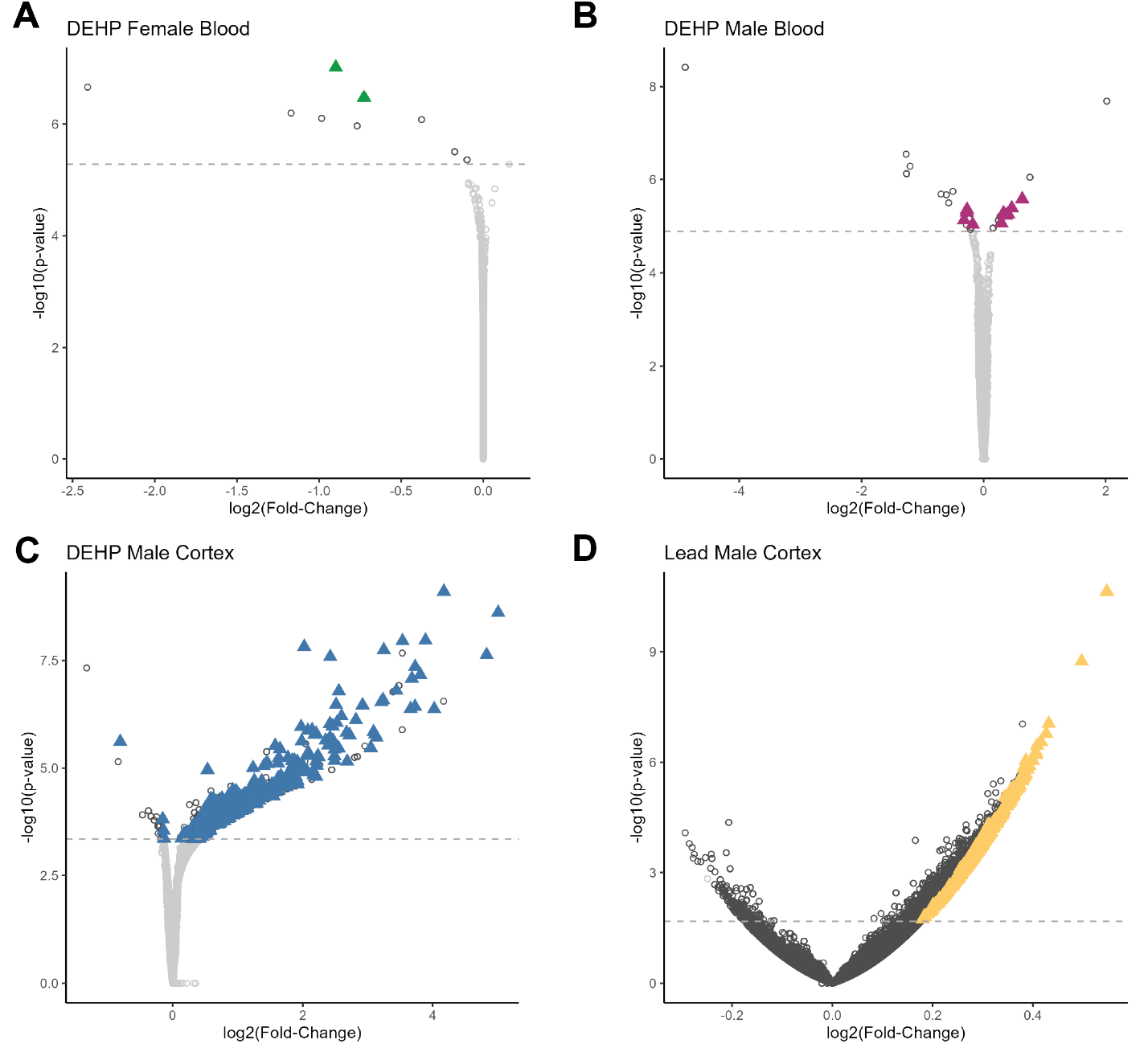


**Supplemental Figure S1**: Volcano plots of DhMRs. Light gray circles represent non-significant regions, dark gray circles represent regions with an FDR<0.15 in the main 100 bp frame analysis, but were not significant with a 500 bp frame, and colored triangles represent regions with an FDR<0.15 in the main 100 bp frame analysis that were also significant with a 500 bp frame.

Supplemental Figure 2: Comparing Fold-Differences

Male Cortex

**Supplemental Figure S2:** Comparing Fold-Differences in Hydroxymethylation in male cortex DhMRs. x-axis highlights differences in lead cortex, y-axis highlights difference in DEHP cortex. DhMRs were not well correlated between exposures.
